# EUFOREA summit in Brussels 2023: *inspiring the future of allergy & respiratory care*

**DOI:** 10.3389/falgy.2023.1236977

**Published:** 2023-07-27

**Authors:** P. W. Hellings, S. Lau, G. K. Scadding, L. Bjermer, V. Backer, A. M. Chaker, D. M. Conti, E. De Corso, Z. Diamant, R. Djukanovic, W. Fokkens, P. Gevaert, C. L. Gray, J. K. Han, L. G. Heaney, H. J. Hoffmann, M. Jesenak, P. Johansen, M. S. Kumaran, M. McDonald, E. Melén, J. Mullol, S. Reitsma, D. Ryan, G. Scadding, P. Schmid-Grendelmeier, T. Teeling, M. Odemyr, U. Wahn

**Affiliations:** ^1^ Department of Otorhinolaryngology-Head and Neck Surgery, University Hospitals, Leuven, Belgium; ^2^ Upper Airways Research Laboratory, Department of Head and Skin, Ghent University, Ghent, Belgium; ^3^ Department of Pediatric Respiratory Medicine, Immunology and Critical Care Medicine, Charité Universitaetsmedizin, Berlin, Germany; ^4^ Department of Allergy & Rhinology, Royal National ENT Hospital, London, United Kingdom; ^5^ Division of Immunity and Infection, University College, London, United Kingdom; ^6^ Department of Respiratory Medicine & Allergology, Institute for Clinical Science, Skane University Hospital, Lund University, Lund, Sweden; ^7^ Department of Otorhinolaryngology, Head & Neck Surgery, and Audiology, Rigshospitalet, Copenhagen University, Copenhagen, Denmark; ^8^ Department of Otorhinolaryngology and Center for Allergy and Environment (ZAUM), TUM School of Medicine, Klinikum rechts der Isar, Technical University of Munich, Munich, Germany; ^9^ The European Forum for Research and Education in Allergy and Airway Diseases Scientific Expert Team Members, Brussels, Belgium; ^10^ Otolaryngology Head and Neck Surgery, A. Gemelli University Hospital Foundation IRCCS, Rome, Italy; ^11^ Department of Respiratory Medicine, First Faculty of Medicine, Charles University and Thomayer Hospital, Prague, Czech Republic; ^12^ Department Clinical Pharmacy and Pharmacology, University of Groningen, University Medical Center Groningen, Groningen, Netherlands; ^13^ Dept of Microbiology Immunology & Transplantation, KU Leuven, Catholic University of Leuven, Leuven, Belgium; ^14^ NIHR Southampton Biomedical Research Centre, Faculty of Medicine, University of Southampton, Southampton, United Kingdom; ^15^ Department of Otorhinolaryngology, Amsterdam University Medical Centres, Amsterdam, Netherlands.; ^16^ Division of Allergy, Department of Paediatrics and Child Health, University of Cape Town, Rondebosch, South Africa; ^17^ Specialist Allergist, Kidsallergy Centre, Cape Town, South Africa; ^18^ Department of Otolaryngology & Head and Neck Surgery, Eastern Virginia Medical School, Norfolk, VA, United States; ^19^ Wellcome-Wolfson Institute for Experimental Medicine, Queens University Belfast, Belfast, United Kingdom; ^20^ Department of Clinical Medicine, University of Aarhus, Aarhus, Denmark; ^21^ Department of Pulmonology and Phthisiology, Department of Pediatrics, Department of Clinical Immunology and Allergology, Jessenius Faculty of Medicine in Martin, Comenius University in Bratislava, University Hospital in Martin, Martin, Slovakia; ^22^ Department of Dermatology, University of Zurich, Zurich, Switzerland; ^23^ Allergy Unit, Department of Dermatology, University Hospital Zurich, Zurich, Switzerland; ^24^ Department of Dermatology, Venereology and Leprology, Post Graduate Institute of Medical Education and Research, Chandigarh, India; ^25^ Mediclinic Sandton, Johannesburg, South Africa; ^26^ Department of Clinical Science and Education Södersjukhuset, Karolinska Institutet and Sachs’ Children and Youth Hospital, Stockholm, Sweden; ^27^ Rhinology Unit and Smell Clinic, ENT Department, Hospital Clínic, FRCB-IDIBAPS, Universitat de Barcelona, CIBERES, Barcelona, Catalonia, Spain; ^28^ Allergy and Respiratory Research Group, Usher Institute of Population Health Sciences and Informatics, University of Edinburgh, Edinburgh, United Kingdom; ^29^ International Primary Care Respiratory Group., Edinburgh, United Kingdom; ^30^ Allergy, Royal Brompton Hospital, London, United Kingdom; ^31^ Christine-Kühne Center for Allergy research and Education CK-CARE, Davos, Switzerland; ^32^ Patient Advisory Board of the European Forum for Research and Education in Allergy and Airway Diseases, Brussels, Belgium

**Keywords:** EUFOREA, asthma, allergic rhinitis, rhinosinusitis, paediatrics, allergen immunotherapy, patient advisory board, pocket guide

## Abstract

In March 2023, the European Forum for Research and Education in Allergy and Airways diseases (EUFOREA) organized its bi-annual Summit in Brussels with expert panel members of EUFOREA, representatives of the EUFOREA patient advisory board, and the EUFOREA board and management teams. Its aim was to define the research, educational and advocacy initiatives to be developed by EUFOREA over the next 2 years until the 10th anniversary in 2025. EUFOREA is an international non-for-profit organization forming an alliance of all stakeholders dedicated to reducing the prevalence and burden of chronic allergic and respiratory diseases via research, education, and advocacy. Based on its medical scientific core competency, EUFOREA offers an evidence-supported platform to introduce innovation and education in healthcare leading to optimal patient care, bridging the gap between latest scientific evidence and daily practice. Aligned with the mission of improving health care, the expert panels of asthma, allergic rhinitis (AR), chronic rhinosinusitis (CRS) & European Position Paper on Rhinosinusitis and Nasal Polyps (EPOS), allergen immunotherapy (AIT) and paediatrics have proposed and elaborated a variety of activities that correspond to major unmet needs in the allergy and respiratory field. The current report provides a concise overview of the achievements, ambitions, and action plan of EUFOREA for the future, allowing all stakeholders in the allergy and respiratory field to be up-dated and inspired to join forces in Europe and beyond.

## Introduction

Chronic airways and allergic diseases are widespread health concerns for millions of people worldwide, reaching epidemic proportions and affecting up to 30% of the general population (1). Asthma, AR and CRS cause significant morbidity, reduced quality of life (2), and substantial healthcare costs (3, 4). The prevalence of chronic airway diseases and allergies has been on the rise globally, and has reached massive numbers, with millions of adults and children affected and in 2017 an increase of 39,8% compared with 1990 was noted (5). The burden of airway diseases and allergies is significant, impacting individuals, families, communities, and healthcare systems (4, 6). People with severe airways diseases and allergies often experience symptoms that negatively impact daily activities, sleep and overall well-being (7, 8). Severe exacerbations of asthma can result in hospitalization, increased use of medications, and even death (9). According to the World Health Organization (WHO), asthma affected an estimated 262 million people in 2019 and caused a staggering 455.000 deaths (10). These conditions also impose a considerable economic burden, including direct costs of medical care, medication expenses, and, equally important, significant indirect costs due to lost productivity and absenteeism from work or school (3).

EUFOREA is an international non-for-profit organization founded in 2015 upon the suggestion of the former European Commissioner of Health Mr. Vytenis Andriukaitis, forming an alliance of multiple stakeholders dedicated to reducing the prevalence and burden of chronic allergic and respiratory diseases through the implementation of optimal patient care via research, educational, and advocacy activities. The following problems have been recognized as obstacles to improvement of care and prevention (1): lack of structural collaboration between larger and smaller organisations with focus on only a segment of the respiratory tract, lack of focus on prevention and optimal care by most scientific international organisations, lack of effective collaboration between patients and specialists from different disciplines within the respiratory and allergy field, lack of joint advocacy initiatives to promote the patients voices both in Europe and beyond, and lack of a truly global patient advisory board with patients suffering from chronic respiratory diseases speaking with one voice to society and health policy makers. Based on its core values of inclusivity and scientific excellence, EUFOREA offers a platform to introduce innovation and education in healthcare leading to optimal patient care, bridging the gap between the latest science and routine clinical practice. The collaboration between primary care physicians, pulmonologists, allergologists, ENT surgeons, paediatricians and patients of the EUFOREA Patient Advisory Board (PAB) reflect the ambition of EUFOREA to be inclusive and multidisciplinary. EUFOREA aims to promote innovation in the diagnosis, treatment, and management of chronic respiratory diseases and allergies, and advocate for policies and regulatory bodies dealing with respiratory health. Following these guidelines, EUFOREA aims mainly at secondary and tertiary prevention true timely diagnosis via screening and timely referral, personalized therapeutic approaches including life-style measures and environmental control with avoidance of triggers. These aspects are included in the different Pocket Guides and algorithms of EUFOREA (11–13).

**Figure 1:**
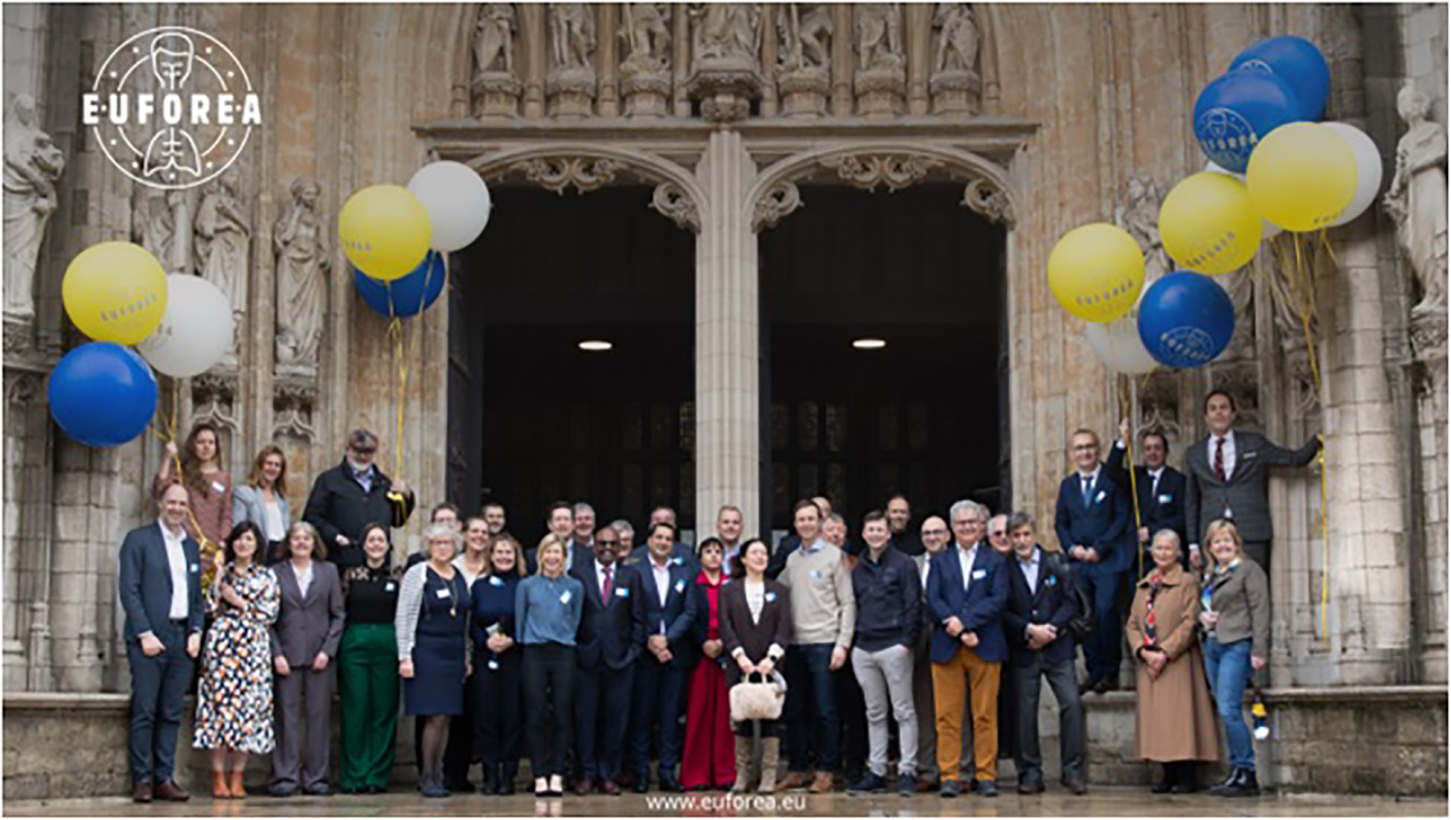
Group picture taken at the 2023 EUFOREA summit in Brussels.

Aligned with the mission of collaboratively improving health care by bridging the gap between the latest updates in science and daily practice, the PAB of EUFOREA and the expert panels of asthma, AR, CRS & EPOS, as well as AIT, were invited to the 2023 Summit of EUFOREA to discuss the future of research, education and advocacy and to propose and prioritise a variety of activities for the coming years to identify unmet needs within the respiratory field (Figure 1).

**Figure 2:**
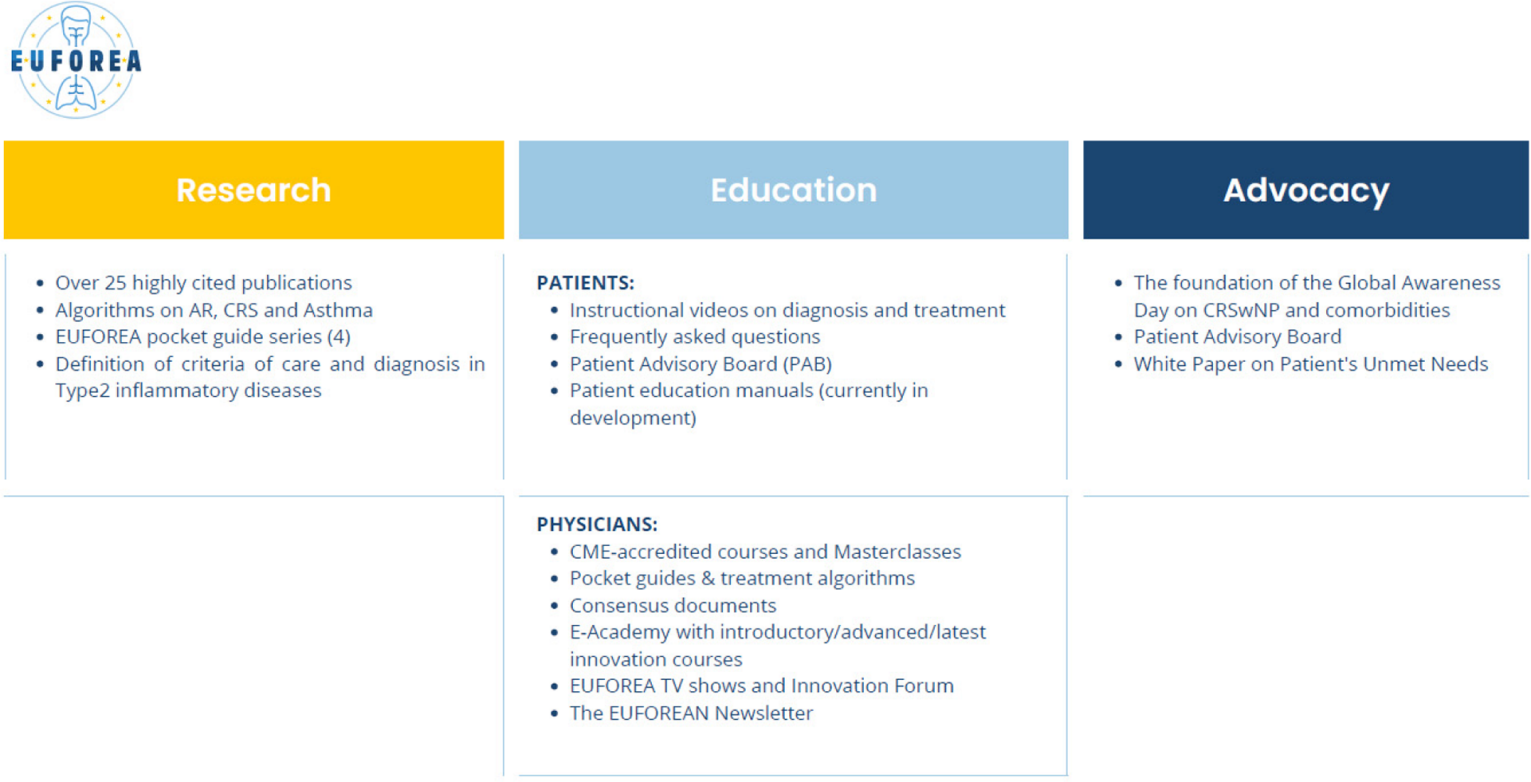
Overview of the EUFOREA achievements.

Since its foundation, EUFOREA has achieved significant milestones that are publicly available on the website with the slogan “*inspiring the future of respiratory care”* (www.euforea.eu, Figure 2). By engaging stakeholders from various disciplines, EUFOREA aims to promote a holistic and multidisciplinary approach to respiratory care and improve respiratory health outcomes for patients.

**Figure 3:**
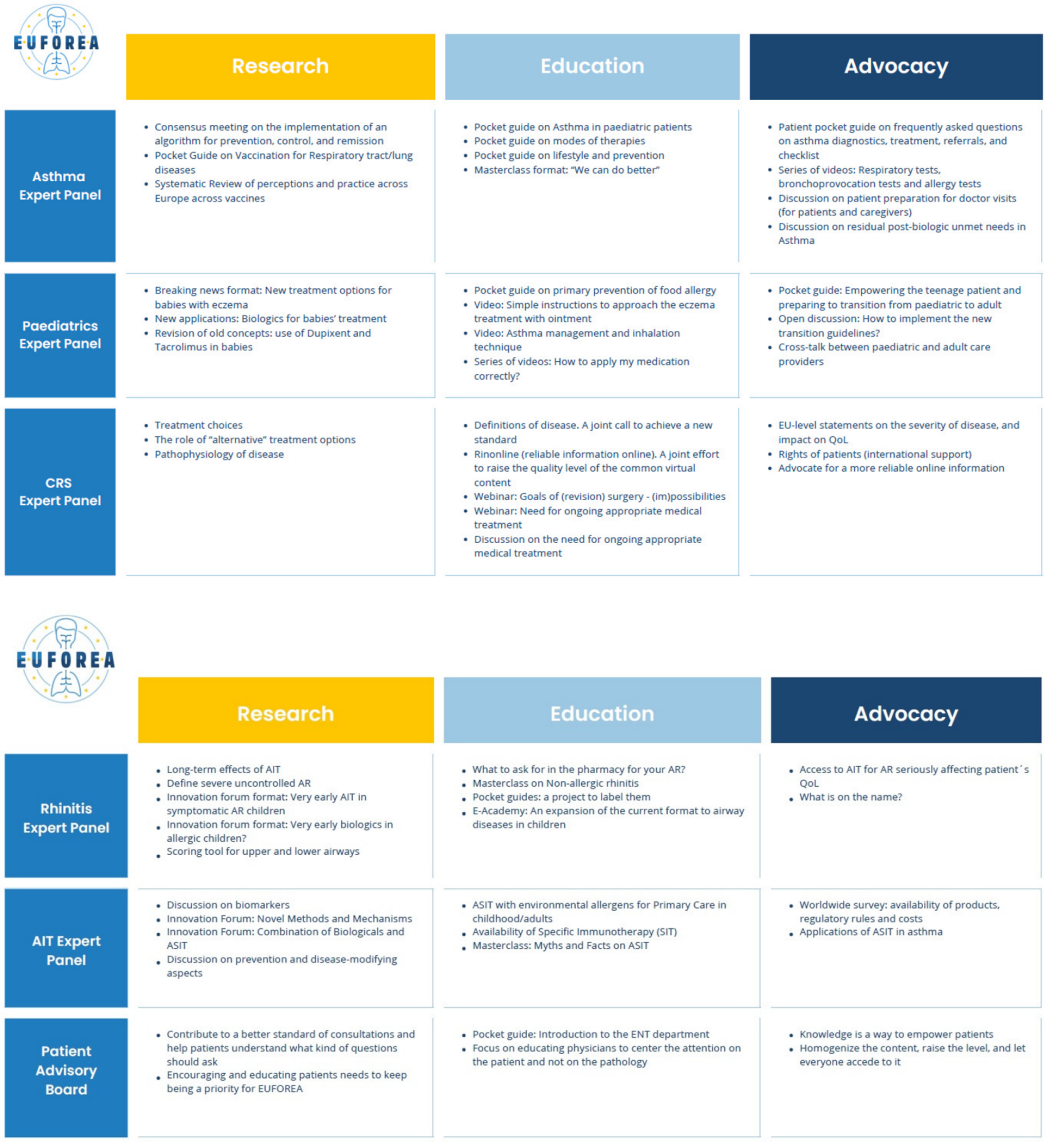
Major unmet needs and proposed activities for the upcoming years in the fields of different expert panels.

The current report provides a concise overview of the achievements, ambitions, and action plan of EUFOREA for the upcoming years discussed during the Summit 2023, allowing all stakeholders in the respiratory and allergy field to be up-dated and ready to join forces to tackle the burden of chronic respiratory diseases in Europe and beyond. All expert panels focussed their discussions on unmet needs in their specific speciality and proposed activities based on unique nature, innovative character, and alignment with the mission and vision of EUFOREA. These criteria guided the panels in assessing the quality, relevance, and potential impact of the proposals in line with the goals and values of EUFOREA. Overall, the discussions underscored the need for increased attention and resources in research, education and advocacy to address unmet needs and promote positive change in various areas within chronic respiratory diseases and allergy. All co-authors of this paper emphasized the importance of collaboration amongst stakeholders, including patients (14), researchers, clinicians, health policy makers, patients, and advocates to work towards meaningful solutions and create a more equitable and inclusive world (Figure 3).

## Research

Two major unmet needs in respiratory research in asthma, allergies and CRS have been recognized as priorities: 1/ to better understand strategies for prevention of chronic respiratory disease, mainly asthma and respiratory diseases, in childhood and adolescence, and 2/ to gain consensus on definitions of and insight into disease modification and remission in people suffering from asthma and/or CRS with Nasal Polyps (CRSwNP). Secondary and tertiary prevention might be achieved via lifestyle counselling and timely implementation of appropriate therapies such as allergen-specific immunotherapy (AIT) in patients with AR and patients with allergen-driven asthma (15), sinus surgery in patients with CRS (16), and target-specific anti-inflammatory biologic therapies. Besides prevention, EUFOREA should be a body through which novel expert consensus can be reached on important clinical aspects of diseases and/or aims of treatment like disease exacerbation, remission, disease modification, and control. The asthma expert panel emphasized the significant differences in vaccination practices for respiratory tract/lung diseases across different countries in Europe due to factors such as national vaccination policies, local epidemiology, and healthcare systems. However, in general, there are several common respiratory tract/lung disease vaccines that are widely used in Europe, including Influenza (Flu), Pneumococcal Vaccine, Pertussis (Whooping Cough) and COVID-19. New vaccines, such as that against RSV, have recently become available. A systematic review on the perceptions and practice across Europe concerning vaccination in patients with respiratory tract diseases with recommendations is needed.

Other expert panels have emphasized the many unmet needs and questions around AIT. By exposing the immune system to allergens to build tolerance (17), AIT has shown promising results in reducing the severity of allergy symptoms and improving patients' quality of life (18). Despite the progress made in AIT, there are still many unmet needs that remain to be addressed. These unmet needs refer to improved patient selection and improved outcomes based on biomarkers and preventive potential, issues related to the long-term effects of AIT, availability of products and/or obstacles for the indication of AIT. Typically, fewer than 7% of patients within the indication for AIT in a population start on AIT (19) and fewer than 30% complete a three-year treatment (20). It has been proposed that a survey is needed to evaluate the long-term effects of AIT in patients who have received it more than a decade ago to assess longevity of benefit. In infancy its use is probably underused and the possibilities of very early AIT in symptomatic AR children was indicated as an important field of interest. Novel methods and mechanisms of administration such as intralymphatic immunotherapy (ILIT), epicutaneous immunotherapy (EPIT), oral immunotherapy (OIT) to modified food, drug and other hypoallergenic products may play an important future role, especially in paediatric patients. EUFOREA looks forward to results of an ongoing international multicenter trial of ILIT performed by its expert panel members (21). Other important unmet needs are the prevention and disease-modifying aspects of AIT and the need for reliable biomarkers in the evaluation of the treatment.

There is still an unmet need for a subgroup with severe uncontrolled allergic rhinoconjunctivitis (often poly-allergic) that still need systemic steroids as they are still not controlled by conventional treatments and where AIT does not cover or control their allergies. For those patients we need to build evidence for the use of biologicals. Also, the combination of AIT with biologics, especially in the paediatric population with severe uncontrolled respiratory and food allergies was mentioned as an area meriting exploration. Following global climate changes, there are also new challenges for both patients and care-givers in relation to a changing allergen panorama (e.g., new allergens), altered pollen seasons (e.g., extreme peaks) and extreme weather conditions (e.g., heat waves) (22).

Building further on the CRS control test being developed and validated by EUFOREA in 2023, a combined upper and lower airway disease control test was considered to be an important unmet need, underscoring the multidisciplinary ambitions of EUFOREA.

While biologics have shown promising results in the treatment of CRSwNP (23) and asthma (24), and recently also of COPD (23, 25, 26), there are still some unmet needs and questions arising from their use. The mechanism of biologics in respiratory diseases are complex and may involve interactions with multiple targets or pathways in the respiratory system. The specific mechanism of action of a given biologic will depend on its intended target and indication and can show anti-inflammatory effect but also disease modification. A very important facet is the prediction of treatment effect in individual patients using biomarkers, as well as real-life data being studied by several research groups affiliated with EUFOREA. Biologics are typically reserved for patients with severe uncontrolled respiratory conditions who have failed first- and second-line treatments. However, determining the optimal patient selection criteria for biologic therapy, such as identifying the patients who are most likely to respond is still an area of ongoing research and, to date, we do not have any biomarkers that could be used to decide which biologic is most likely to be effective in which patient. The ongoing European registries and the EUFOREA registry on real-world efficacy of biologics for the indication of CRSwNP might shed interesting light on the efficacy, size of effect, speed of action, safety, and outcomes of switching from one to another biologic. It is often unclear which biologic should be used depending on disease characteristics and co-morbidities. Criteria for switching between biologics should be established based on emerging evidence. For some biologics it has been shown that the dosing interval can be prolonged without losing control of disease (27), an important factor when considering the health economics of these costly treatments and a significant pillar of personalised medicine. However, for most biologics the options of dose prolongation have not been evaluated. While clinical trials, ongoing post-marketing studies and surveillance have shown biologics to be generally safe, more research is needed to determine any potential risks or side effects associated with their long-term use, especially in infancy and during pregnancy. In particular, the possibilities and limitations of biologics in the treatment of children with severe airway diseases remain underexplored.

## Education

Education is one of the key pillars of EUFOREA. Education encompasses both patients and physicians, as it involves providing information and knowledge to both groups to improve healthcare outcomes. In 2022, over 15,000 physicians attended CME-accredited courses or online educational materials provided by EUFOREA, with various patient instructional/educational videos being viewed more than 225,000 times. The expert panels emphasized the importance of readily available and easily accessible educational material. The development of pocket guides with simplified treatment algorithms for CRS (11), AR (12, 13) and asthma has been shown to be an important tool to educate both physicians and patients about lifestyle, prevention, treatment options and expected outcomes of different strategies. The ideal pocket-guide should be concise and should contain all relevant information allowing all stakeholders dealing with respiratory care to understand the key considerations of care. Also, contemporary ways of communication via video, webinars, YouTube and/or other social media can be appropriate depending on the target population. When addressing proper diagnoses and/or correct use of treatment, visual information can be vital. EUFOREA uses many different ways to reach out to patients and caregivers, both written and audio-visual.

For physician education, masterclasses and the recently launched e-academy have been developed with novel materials aimed at meeting the needs of healthcare providers with different levels of knowledge and specialization.

The most important set of information proposed by the patient representatives is a pocket-guide written in non-technical terms, to facilitate patients and physicians to enable an optimal conversation, suggesting suitable questions to make the best use of the consultation. A well-informed patient is prepared with sound information about his/her disease, has a good overview of his/her own medical history, including use of medication and relevant symptoms and can provide an accurate and comprehensive synopsis during the visit. With appropriate knowledge about the (im)possibilities of certain treatments patients and their caregivers can make better choices for an optimal management approach. Lay language pocket guides can help patients be better prepared to collaborate in the decision-making process. Besides the online available pocket-guides, the field is still open and some topics were suggested for further development: specific information on vaccination for respiratory tract/lung diseases in adults and children, asthma in paediatric patients, asthma therapy including one on specific therapies such as biologics/AIT and lifestyle advice and the prevention of asthma. The importance of understanding the chronicity of disease and thus the need for ongoing and correct use of medication needs to be stressed.

Primary prevention of food allergy in agreement with international guidelines including information on early introduction of family foods are issues to be addressed for babies and their care-givers.

It was observed that a significant part of disease information available online is inadequate or wrong. There is a substantial need for reliable information online. Important questions are how the patient is able to winnow out the wheat from chaff. It was proposed to investigate available information online, with specific emphasis on frequently visited sites like Wikipedia and improve the information there as much as possible. The excellent information on the EUFOREA website needs to be better promoted to encourage patients to find it more easily.

Some diseases and symptoms, like loss of smell, are under-empathized or under-taught, resulting in failure to make accurate diagnoses. Also, allergic diseases in children are often erroneously considered to have an infectious origin and as a consequence are mis-treated. Caregivers may have difficulties in the understanding and management of exacerbations and their usually viral origin. For more condition- specific education, the expert groups felt a need for more and better education on treatment and medication such as on topical corticosteroid safety, compared to oral corticosteroid toxicity, the options of AIT or biologics, and the possibilities to modify disease or even achieve remission. For surgical treatment the goals of (revision) surgery and the (im)possibilities to improve certain symptomatology should be clear to both the surgeon and the patient.

## Advocacy

Advocacy plays a critical role in promoting positive social change and addressing issues that impact individuals and communities (7). Despite their high prevalence, many people unaware of conditions like AR, CRS or asthma, and the impact of diagnostic neglect on the quality of life and loss of preventive potential. The symptoms of CRS, such as chronic nasal congestion, facial pain or pressure, and loss of smell, have a significant negative impact on quality of life, particularly on a vital necessity, restful sleep. Furthermore, because the symptoms of AR and CRS are similar to those of other conditions such as a common cold, they may be misdiagnosed or overlooked. This can lead to delays in treatment and a further impact on a person's quality of life. Also, other airway diseases and allergies are often underdiagnosed and undertreated. Therefore, increasing awareness of airway diseases is essential to ensure that individuals who are experiencing these symptoms receive an accurate diagnosis and appropriate treatment. EUFOREA has addressed the unmet needs of patients with CRSwNP with the Global Awareness Day of CRSwNP in the Parliament in 2022 and with over 10 non-for-profit organizations joining in 2023 (5)**.**

Raising awareness is not only an important issue at the level of the general public but especially important in relation to healthcare decision-makers and payers. EUFOREA provides statements on the severity of disease, and impact on QoL, organizes discussions between patients and policy makers and health care funders to explain the impact on QoL and, if needed, explains to patients their rights to necessary care (4). The large inequalities within the EU concerning the availability and reimbursement of biologics, especially for CRSwNP, are confusing and unjustified. A worldwide survey on the availability of products, regulatory rules and costs would expose these differences and open the discussion on the significant indirect cost of airway diseases that are often unrecognized and can potentially be significantly reduced by (often expensive but very effective) treatment. On the other hand, the arrival of biologics has been a great step forward in the treatment of type 2 upper and lower airway disease (28), but those drugs are not the solution for all airway diseases. For other (non-type 2) airway diseases management options are still limited and improvements with new treatments are warranted. Dialogue between pharmaceutical companies and funders is critical to offer patients access to effective, novel, albeit costly, treatment.

Not all patients have the strength to fight for their rights. Education of patients is a way to empower them, and EUFOREA plays an important role in improving knowledge of patients and healthcare providers alike. The health-literate patient is a better partner during a consultation and better manages his/her own condition. Adequate, accurate, easily accessible and understandable (online) information is one of the most powerful tools to empower patients. Teenagers who are transitioning into the adult-type healthcare system, in which their disease is often taken care of by several different specialists rather than one overseeing specialist, are a sensitive and delicate group of patients who need help and empowerment in navigating their new circumstances. Also, it is very important that such a transition is well- guided and facilitated. EUFOREA can help in developing guidance in this transition and define guidelines as to what steps have to be taken in the transition process. Not only teenagers, but also adult patients often have difficulties asking the right questions and knowing what the options are in the management of their disease. Empowering patients to know what to ask and to know what to ask for cannot be emphasized enough. EUFOREA wants to play an important role in the process. The near future holds several initiatives through which EUFOREA will take an active role in contributing to the defence and support of patients and their rights. Slogans such as “every asthma patient should breathe through their nose” and “type 2 inflammation is not just rhinitis or asthma” are just the starting point for EUFOREA.

## Summary

The high prevalence and major socio-economic impact of chronic inflammatory airway diseases and allergies require an inter-academic and multi-stakeholder approach for the successful implementation of prevention and management strategies leading to cost- savings and reduction in the burden of disease. EUFOREA will continue its mission to call for action by all stakeholders to implement advocacy and precision medicine as the tools to arrest the epidemic of chronic respiratory diseases (CRD).

In Europe, there is an urgent need to join forces in the education of patients and medical care providers on management and prevention strategies, and to call for political action supported by all European academic stakeholders involved in the care of CRD.

An overall summary of the content expressed in this article is shown in Figure 3.

## Data Availability

The raw data supporting the conclusions of this article will be made available by the authors, without undue reservation.
